# Ultrasound-Assisted Extraction of Bioactives from *Spirulina platensis*: Optimization and Prediction of Their Properties Using Near-Infrared Spectroscopy Coupled with Artificial Neural Network Modeling

**DOI:** 10.3390/foods14193358

**Published:** 2025-09-28

**Authors:** Blaženko Marjanović, Tea Sokač Cvetnić, Davor Valinger, Jasenka Gajdoš Kljusurić, Tamara Jurina, Maja Benković, Ana Jurinjak Tušek

**Affiliations:** Faculty of Food Technology and Biotechnology, University of Zagreb, Pierottijeva 6, 10000 Zagreb, Croatia; bmarjanovic@pbf.hr (B.M.); tsokac@pbf.hr (T.S.C.); davor.valinger@pbf.unizg.hr (D.V.); tamara.jurina@pbf.uniz.hr (T.J.); maja.benkovic@pbf.unizg.hr (M.B.)

**Keywords:** extraction optimization, chemometrics, non-destructive analysis, functional ingredients, quality control

## Abstract

This study optimizes the ultrasound-assisted extraction (UAE) of bioactive compounds from *Spirulina platensis* and develops a rapid, non-destructive analytical method. A Box–Behnken design and desirability function were used to find the optimal extraction conditions to simultaneously maximize total polyphenols, proteins, C-phycocyanin, and antioxidant activity. The optimal conditions were a solid-to-liquid ratio of 35 g/L, a time of 20 min, a pH of 10, and a temperature of 45 °C. Independent validation confirmed the model’s reliability, with experimental results closely matching predicted values. Furthermore, Near-Infrared (NIR) spectroscopy, combined with Artificial Neural Networks (ANNs), was explored as a predictive tool. The models, particularly those based on a semi-process NIR spectrometer, showed excellent predictive capabilities for key physicochemical properties, with an RPD of 3.9064 for L* and 2.8351 for TDS. This research establishes a highly reproducible and scalable extraction protocol, complemented by a fast and accurate analytical method, providing a significant advancement for the industrial application and quality control of *Spirulina platensis* extracts.

## 1. Introduction

Microalgae have garnered significant attention as a sustainable source of a wide array of high-value bioactive compounds, including proteins, polysaccharides, lipids, pigments, and antioxidants. Among these, *Spirulina platensis* stands out as a “superfood” with a rich nutritional profile and a long history of use in human and animal nutrition [1,2,3]. The commercial value of *Spirulina* is largely attributed to its wealth of bioactive components, such as C-phycocyanin, a blue pigment-protein complex with potent antioxidant, anti-inflammatory, and neuroprotective properties, and phenolic compounds, which contribute significantly to its overall antioxidant capacity [2,4,5,6]. The efficient extraction of these compounds is a critical step in their industrial application. Traditional extraction methods often rely on organic solvents, are time-consuming, and can be energy-intensive, leading to low yields and potential degradation of sensitive compounds [7,8,9]. Consequently, there is a growing need for more efficient, green, and scalable extraction techniques [8,10,11,12,13].

Ultrasound-assisted extraction (UAE) has emerged as a promising alternative for the recovery of bioactive compounds from plant and algal biomass [8,14,15,16,17]. This technique uses high-frequency sound waves to create microscopic bubbles (cavitation) in the solvent. The collapse of these bubbles generates localized high pressure and temperature, disrupting cell walls and enhancing the release of intracellular components into the solvent. Compared to conventional methods, UAE offers several advantages, including reduced extraction time, lower solvent consumption, and improved yields [18,19]. Optimizing the condition of the UAE, such as the solid-to-liquid ratio, solvent pH, temperature, and extraction duration, is crucial for maximizing the yield and quality of the final extract [20]. The complex interplay of these variables necessitates a systematic approach to identify the optimal conditions, which is where methods like Response Surface Methodology (RSM) prove invaluable [21]. RSM allows for the modeling of the extraction process and prediction of the response (e.g., total protein content, antioxidant activity) based on the input variables.

While process optimization is essential, a significant bottleneck in the industrial production of microalgal extracts is the lack of a rapid and non-destructive method for real-time quality control. Current methods for analyzing key properties such as protein content, C-phycocyanin, total phenolics, and antioxidant activity are based on well-established assays including Bradford, Folin–Ciocalteu, DPPH, and FRAP. These techniques are widely recognized, relatively fast, and provide validated reference data that remain essential for accurate quantification. However, they are laboratory-based, require sample preparation and reagents, and are inherently destructive, which limits their suitability for continuous or on-line monitoring in dynamic extraction processes. Near-Infrared (NIR) spectroscopy, a vibrational spectroscopy technique, offers a compelling solution to this challenge [22]. NIR spectroscopy measures the absorption of light in the Near-Infrared region (780–2500 nm), which corresponds to the overtones and combination bands of the fundamental vibrations of C-H, N-H, and O-H bonds. These bonds are the fundamental building blocks of key biomolecules such as proteins, lipids, and carbohydrates, making the NIR spectrum a unique chemical “fingerprint” of a sample [23,24,25,26]. The technique is fast, non-destructive, requires little to no sample preparation, and can be easily automated for online applications. The complexity of NIR spectra, however, requires the use of multivariate data analysis, or chemometrics, to extract meaningful information and build predictive models.

The successful application of NIR spectroscopy for predicting complex sample properties depends heavily on the quality of the chemometric models. Traditional methods like Partial Least Squares (PLS) regression have been widely used, but they assume a linear relationship between spectral data and sample properties [27]. However, the relationships between the NIR spectrum of a complex biological matrix like a *Spirulina* extract and its biochemical properties are often highly non-linear [28]. This is where Artificial Neural Networks (ANNs) offer a significant advantage. ANNs are powerful machine learning algorithms capable of learning and modeling highly complex, non-linear relationships within a dataset [29,30]. By training an ANN on a large dataset of paired NIR spectra and reference values (e.g., from Bradford or FRAP assays), a robust predictive model can be developed.

The novelty of this research lies in its integrated approach, which combines three advanced methodologies to address the critical gaps in *Spirulina* extract production: ultrasound-assisted extraction optimization, NIR spectroscopy for rapid analysis, and ANN modeling for enhanced prediction. This research aims to provide a proof-of-concept for a new paradigm in microalgal extract production, moving away from slow, lab-based analysis towards a real-time, sensor-driven approach. By demonstrating the high predictive power of NIR spectroscopy coupled with ANN modeling for *Spirulina* extracts, this study paves the way for the development of smart, automated extraction systems capable of continuous process monitoring and optimization. The results will be of great interest to the nutraceutical, food, and cosmetic industries, accelerating the adoption of sustainable and efficient production methods for high-value microalgal compounds.

## 2. Materials and Methods

### 2.1. Algae and Chemicals

Dried *Spirulina platensis* powder was sourced from Nutrigold (Zagreb, Croatia). Analytical-grade chemicals and reagents were used throughout the study. Several key compounds, including TPTZ (2,4,6-tris(2-pyridyl)-s-triazine), gallic acid (98%), iron (II) sulfate heptahydrate, DPPH (1,1-diphenyl-2-picrylhydrazyl), Trolox (6-hydroxy-2,5,7,8-tetramethylchromane-2-carboxylic acid), and sodium chloride, were supplied by Sigma-Aldrich Chemie (Steinheim, Germany). Additional chemicals such as hydrochloric acid (30%), iron (III) chloride hexahydrate, sodium carbonate, and sodium chloride were obtained from Gram-Mol d.o.o. (Zagreb, Croatia). Sodium acetate trihydrate was purchased from J.T. Baker (Deventer, The Netherlands), while sodium hydrogen carbonate was sourced from Franck (Zagreb, Croatia). Kemika d.d. (Zagreb, Croatia) provided the Folin–Ciocalteu reagent, disodium hydrogen phosphate, and sodium dihydrogen phosphate dihydrate. Acetic acid was procured from T.T.T. d.o.o. (Sveta Nedjelja, Croatia), and methanol was supplied by Carlo Erba Reagents S.A.S. (Val de Reuil, France).

### 2.2. Methods

#### 2.2.1. Ultrasound-Assisted Extraction of Bioactive Compounds and Process Optimization by Response Surface Methodology

The extraction of bioactive compounds from *Spirulina platensis* was performed using an ultrasonic bath (DT 103 H, Bandelin Electronic, Berlin, Germany). A precisely weighed amount of dried algal biomass was dispersed in an appropriate volume of deionized water in a 50 mL flask, with the solvent pH adjusted using a 914 pH/Conductometer (Metrohm AG, Herisau, Switzerland). 0.1 M HCl and 0.1 M NaOH were used to adjust the solvent. These dilute concentrations allow for precise, incremental changes to the pH without causing rapid shifts that could denature sensitive bioactives. The suspensions were subjected to ultrasound-assisted extraction under varying process conditions, followed by centrifugation at 8000 rpm for 10 min. The supernatant obtained was used for further analysis, and all samples were stored at 4 °C until analysis. Extraction conditions were selected based on the relevant scientific literature [31,32,33].

To optimize the extraction efficiency, Response Surface Methodology (RSM) was applied using a Box–Behnken design in Statistica 14.0 (TIBCO Software Inc., Palo Alto, CA, USA). Four independent variables were studied at three levels each: solid-to-liquid ratio (15, 25, 35 g/mL), extraction time (20, 40, 60 min), pH (7, 8.5, 10), and temperature (25, 35, 45 °C). The design generated 30 experimental runs (Table 1).

The fundamental idea of RSM is to model the relationship between influential factors and the response variable using an appropriate response function. Since the exact form of this relationship is generally unknown, a suitable approximation function—usually a lower-order polynomial—is selected within the chosen range of independent variables. Typically, either a first-order (linear) model or a second-order (quadratic) model is employed when nonlinearity is present, as follows [21]:

(1) $$y=\beta_{0}+\beta_{1}\cdot x_{1}+\beta_{2}\cdot x_{2}+\ldots +\beta_{k}\cdot x_{k}+\varepsilon ,$$

(2) $$y=\beta_{0}+\sum_{i=1}^{k}\beta_{i}\cdot x_{i}+\sum_{i=1}^{k}\beta_{ii}\cdot x_{i}^{2}+\sum_{i<j}\sum_{j=2}\beta_{ij}\cdot x_{i}\cdot x_{j}+\varepsilon ,$$

here, *y* is the dependent variable (response), *x_i_* are the independent variables, *β* represents the model coefficients, and *ε* is the residual error. The model coefficients are estimated by minimizing the sum of squared deviations, followed by validation on an independent dataset.

#### 2.2.2. Physical Properties of Extracts

The physical properties of the aqueous extracts, including electrical conductivity and total dissolved solids content, were determined using a conductometer (SevenCompact S230, Mettler-Toledo International Inc., Greifensee, Switzerland).

#### 2.2.3. Color Determination of *Spirulina platensis* Extract Samples

The color of the *Spirulina platensis* extract samples was measured using a colorimeter (PCE-CSM3, PCE Instruments, Meschede, Germany). The analysis was conducted based on the Hunter Lab color space system, where

L* represents the lightness of the sample (ranging from 0 = black to 100 = white),
a* indicates the red-green axis (negative values = green, positive values = red), and
b* corresponds to the blue-yellow axis (negative values = blue, positive values = yellow).

Based on the obtained a* and b* values, the hue angle (color tone) and chroma (color intensity) were calculated using the following equations:

(3) $$Hue=\arctan\left(\frac{b^{*}}{a^{*}}\right)$$

(4) $$C=\sqrt{{\left(a^{*}\right)}^{2}+{\left(b^{*}\right)}^{2}}$$

The hue angle (Hue) defines the shade or tone of the color, while chroma (C) represents the intensity or saturation of the color.

#### 2.2.4. Measurement of Protein Concentrations in the Extracts

Protein content was determined using the Bradford colorimetric assay, with bovine serum albumin (BSA) serving as the calibration standard at a concentration of 1 mg/mL [34]. A 0.5 mL aliquot of each sample was combined with 0.5 mL of Bradford reagent. Following a 30 min incubation period, absorbance was recorded at 595 nm using a spectrophotometer (Biochrom Libra S11, Biochrom, Cambridge, UK). Results were expressed in mg/L. All measurements were performed in five replicates, and the data are reported as mean values with corresponding standard deviations.

#### 2.2.5. Measurement of C-Phycocyanin Content

Prepared protein extracts were subjected to centrifugation, after which the optical density of the supernatant was measured spectrophotometrically at wavelengths of 620 nm and 652 nm. The concentration of C-phycocyanin (CPC) was calculated according to Equation (5), as described by Vernès et al. [35], as follows:

(5) $$CPC=\frac{{OD}_{620}-0.474\cdot {OD}_{652}}{5.34}$$

where

CPC—C-phycocyanin concentration (mg/mL);
OD_620_—optical density at 620 nm;
OD_652_—optical density at 652 nm.

#### 2.2.6. Total Phenolic Content of the Extracts Measurement

The total polyphenol content (TPC) in *Spirulina platensis* extracts was assessed using a spectrophotometric method based on the procedure outlined by Pinelo et al. [36], which involves a colorimetric reaction between phenolic compounds and the Folin–Ciocalteu reagent. In short, 7.9 mL of distilled water was combined with 500 µL of Folin–Ciocalteu reagent (diluted with water in a 1:2 ratio) and 100 µL of the sample. The reaction was initiated by the addition of 1.5 mL of 20% sodium carbonate solution. After a 2 h incubation in the dark, the absorbance of the reaction mixture was measured at 765 nm using a spectrophotometer (Biochrom Libra S11, Biochrom, Cambridge, UK). Total polyphenol concentrations were calculated from a gallic acid calibration curve in the range of 0–500 mg/L. All measurements were performed in five replicates, and the results are expressed as milligrams of gallic acid equivalents (GAE) per gram of dry weight (mg_GAE_/g_dw_).

#### 2.2.7. Determination of Antioxidant Capacity Using the DPPH Method

The antioxidant capacity of the samples was evaluated using the DPPH radical scavenging method, as described by Brand-Williams et al. [37]. A methanol DPPH solution (0.094 mmol/L) was prepared, and 100 µL of the sample was mixed with 3.9 mL of the DPPH solution. The mixture was vortexed (Neuation iSwix VT, Neuation Technologies, Gujarat, India) and incubated in the dark for 30 min. Absorbance was then measured at 515 nm against a blank (methanol instead of sample). Antioxidant activity was quantified using a Trolox calibration curve (0–1 mmol/L), and results were expressed as mmol Trolox equivalents per gram of dry weight (mmol_TE_/g_dw_).

#### 2.2.8. Determination of Antioxidant Activity Using the FRAP Method

The FRAP (ferric reducing antioxidant power) assay was used to evaluate the antioxidant activity of the samples, based on the reduction in the Fe^3+^–TPTZ complex to the blue-colored Fe^2+^–TPTZ form, with absorbance measured at 593 nm [38]. The FRAP reagent was freshly prepared by mixing 25 mL of 300 mmol/L acetate buffer, 2.5 mL of 10 mmol/L TPTZ solution, and 2.5 mL of 20 mmol/L FeCl_3_·6H_2_O solution. A volume of 50 µL of the sample was added to 950 µL of the FRAP reagent, and after 4 min of incubation, absorbance was measured. Antioxidant capacity was calculated using a calibration curve of FeSO_4_·7H_2_O (0–1 mmol/L), and results were expressed as mmol FeSO_4_·7H_2_O per gram of dry weight (mmol_FeSO_4_·7H_2_O_/g_dw_).

#### 2.2.9. NIR Spectroscopy

NIR spectra of *Spirulina platensis* extracts were recorded using two NIR instruments: (i) semi-process NIR spectrometer (NIR-128-1.7-USB/6.25/50 μm, Control Development Inc., South Bend, IN, USA), which records absorbance in the wavelength range of λ = 904–1699 nm. Spectra were analyzed using the Control Development Spec 32 software (Control Development Inc., South Bend, IN, USA) and (ii) a Benchtop NIR spectrometer (AvaSpec-NIR256-2.5-HSC-EVO, Avantes Inc., Lafayette, LA, USA), which records absorbance in the wavelength range of λ = 1000–2500 nm. Spectra were analyzed using AvaSoft-Basic software (Avantes Inc., Lafayette, LA, USA). For each prepared extract, NIR spectra were recorded five times.

#### 2.2.10. Basic Statistical Analysis and Correlation Matrix

All measurements of *Spirulina platensis* extract properties were performed in triplicate. Basic statistical analysis (including the calculation of mean values, standard deviations, ranges, and coefficients of variation) was conducted using the Statistica 14.0 software package (TIBCO Software Inc., Palo Alto, CA, USA). Correlations between extraction conditions and the properties of *Spirulina platensis* extracts were analyzed using Spearman correlation matrices in the same software package. The Spearman matrix was used because the analysis indicated that the data did not follow a normal distribution.

#### 2.2.11. Principal Component Analysis (PCA) of Continuous NIR Spectra

The collected NIR spectra of *Spirulina platensis* extracts, obtained during the optimization experiments of extraction conditions, were analyzed using Principal Component Analysis (PCA) in the Statistica 14.0 software package (TIBCO Software Inc., Palo Alto, CA, USA). PCA is one of the simplest multivariate statistical methods and can be defined as a tool for identifying similarities and differences among data. The goal of PCA is to construct a new coordinate system with a reduced number of dimensions compared to the original data, emphasizing the main sources of variability within the dataset.

#### 2.2.12. Artificial Neural Network (ANN) Modeling

Artificial Neural Network (ANN) modeling was used to individually predict the properties of *Spirulina platensis* extracts, including total dissolved solids, total phenolic content, antioxidant activity determined by DPPH and FRAP methods, total protein concentration, and C-phycocyanin concentration, based on raw NIR spectra collected from both previously described NIR instruments. The ANN models, specifically multilayer perceptron networks (MLP), were developed using the Statistica 14.0 software package (TIBCO Software Inc., Palo Alto, CA, USA).

The ANN architecture consisted of three layers: input, hidden, and output. The input layer included 5 neurons representing the coordinates of the first five principal components obtained from PCA of NIR spectra. These five principal components explained more than 99.99% of the data variability and were selected as model inputs to reduce dimensionality and noise. The hidden layer contained a variable number of neurons, ranging from 4 to 13, which was randomly selected by the algorithm in order to explore different network complexities and avoid overfitting. Each neuron in the hidden layer was fully connected to the preceding input layer, and the weights of these connections were initialized randomly. The output layer consisted of a single neuron, corresponding to the predicted response variable. Activation functions for the hidden and output layers were randomly chosen from four candidates: identity, logistic (sigmoid), hyperbolic tangent (tanh), and exponential functions. This randomized selection allowed the model to capture both linear and non-linear relationships between the input features and the output. To further improve model generalization, the training process included multiple iterations with different hidden-layer sizes and activation function combinations.

The network was trained using a backpropagation algorithm with gradient-based optimization, adjusting weights iteratively to minimize the prediction error. Early stopping criteria and cross-validation were applied to prevent overfitting and to ensure robust model performance across independent datasets.

The dataset used for ANN development had dimensions of 90 × 11, where 90 rows represented the number of *Spirulina platensis* extracts, 5 columns corresponded to PCA coordinates, and the remaining 6 columns contained measured sample properties. For each output variable, a total of 2000 networks were developed. Model training was performed using the backpropagation algorithm with the sum of squares as the error function. The dataset was randomly split into calibration and prediction sets at a 70:30 ratio. Within the calibration set, 70% of data were used for training, 15% for testing, and 15% for validation. The applicability of the developed calibration models was evaluated by the coefficient of determination for calibration (*R*^2^_cal_), adjusted coefficient of determination for calibration (*R*^2^_cal,adj_), and root mean square error of calibration (RMSEC). The predictive performance was assessed using the coefficient of determination for prediction (*R*^2^_pred_), adjusted coefficient of determination for prediction (*R*^2^_pred,adj_), root mean square error of prediction (RMSEP), standard error of prediction (SEP), ratio of performance to deviation (RPD), and range error ratio (RER) [39]. The same methodology was applied for analysis of NIR spectra collected with both NIR instruments.

## 3. Results and Discussion

### 3.1. Physicochemical Characteristics of Spirulina platensis Blue–Green Algae Extracts Obtained Using Ultrasound-Assisted Extraction

*Spirulina platensis* is highly suitable for the extraction of bioactive compounds because of its rich and diverse biochemical composition [2,40]. As a blue–green microalga, it contains high amounts of proteins. It is also one of the best natural sources of C-phycocyanin, a blue pigment-protein complex with strong antioxidant, anti-inflammatory, and coloring potential, widely applied in the food and pharmaceutical industries. In addition to proteins and pigments, *Spirulina* is abundant in polyphenols, which contribute to its bioactivity and health-promoting properties [41]. These compounds are linked to antioxidant, antimicrobial, anti-cancer, and immune-supporting effects, making *Spirulina* an attractive raw material for nutraceuticals and functional foods.

In this work, potential of ultrasound-assisted extraction for the extraction of bioactive forms from *Spirulina plantains* was analyzed. The physical properties of *Spirulina platensis* blue-green algae extracts prepared by ultrasound-assisted extraction display considerable variation depending on the experimental conditions applied. The results presented in Supplementary Table S1 cover a broad range of physicochemical properties, including total dissolved solids (TDS), electrical conductivity (G), and a set of colorimetric characteristics described by the CIE Lab* system along with hue and chroma values. Together, these variables provide insight into both the compositional attributes of the extracts and their visual qualities, which are important for evaluating extraction efficiency as well as potential applications of the product.

TDS values varied widely from 654 to 1600.5 mg/L, suggesting ultrasound treatment had a differing effect on the release of intracellular components. Higher TDS values, as seen in Exp. 3, 19, and 21 (all over 1500 mg/L) indicate more efficient extraction of soluble substances like proteins and pigments. Conversely, low TDS values in Exp. 11 and Exp. 5 points to reduced solubilization. Electrical conductivity mirrored this trend, ranging from 1348.5 to 3230 µS/cm and showing a strong correlation with TDS, confirming the release of ionic species. The L* color coordinate, which measures lightness, was more stable, with most samples being dark (L* ~41–43). A notable exception was Exp. 5 (L* = 54.93), which produced a distinctly lighter extract. The a* and b* coordinates, representing the red-green and yellow-blue axes, respectively, were generally low, indicating a predominantly bluish-green hue. However, the hue angle showed significant variation (2.12 to 68.25), suggesting shifts in the pigment profile. Chroma values, indicating color saturation, were low overall, with Exp. 11 and Exp. 1 showing the highest saturation and Exp. 4 the lowest. This data reveals a strong link between solubility parameters (TDS, EC) and the visual characteristics of the extracts. Ultrasound-assisted extraction can produce both consistent results and marked outliers, with specific experiments like Exp. 5, 11, and 4 demonstrating unique pigment and solubility behaviors.

The chemical properties of *Spirulina platensis* blue-green algae extracts prepared by ultrasound-assisted extraction demonstrate notable variability across the different experimental runs, as summarized in Table 2. The total polyphenol concentration (TPC) varied widely, ranging from 5.789 mg_GAE_/g_dw_ in Exp. 1 to 30.051 mg_GAE_/g_dw_ in Exp. 10. On average, most experiments yielded TPC values between 8 and 15 mg_GAE_/g_dw_, with only a few cases reaching higher concentrations. Experiments 10 and 21 were outliers, providing 30.051 and 21.570 mg_GAE_/g_dw_, respectively, suggesting that specific sonication conditions may greatly enhance polyphenol recovery. By contrast, extracts such as Exp. 1 and Exp. 12, both below 8 mg_GAE_/g_dw_, indicate lower efficiency in liberating phenolic compounds. Similar results were presented by Kamil et al. [42], where 10.517  mg_GAE_/g_dw_, and Martinis et al. [43] where 36.50 mg_GAE_/g_dw_, was obtained in the UAE using deep eutectic solvents. The moderate values observed across the majority of samples confirm that ultrasound treatment successfully extracts polyphenolic compounds but that optimization is required to maximize yields. Antioxidant activity, measured by the DPPH assay, demonstrated important differences between experiments. The range extended from 0.003 mmol_TE_/g_dw_ in Exp. 1 to 0.030 mmol_TE_/g_dw_ in Exp. 13. Most experiments fell between 0.010 and 0.020 mmol_TE_/g_dw_, indicating relatively modest but consistent radical scavenging capacity across the extracts. Higher antioxidant activity was recorded in Exp. 11 (0.027), Exp. 14 (0.028), and Exp. 13 (0.030), which may be related to higher concentrations of antioxidant pigments or polyphenols in these samples. On the other hand, Exp. 1 and Exp. 20, with values of 0.003 and 0.007, displayed the weakest DPPH activity. These variations point toward differences in the qualitative composition of antioxidant molecules in addition to their concentration. The FRAP assay, which measures ferric reducing antioxidant power, showed values ranging from 0.003 mmol_FeSO_4_·7H_2_O_/g_dw_ in Exp. 10 to 0.034 mmol_FeSO_4_·7H_2_O_/g_dw_ in Exp. 16. While most results clustered between 0.006 and 0.015, Exp. 16 clearly stood out with the highest reducing capacity. Other notable cases included Exp. 22 (0.017) and Exp. 17 (0.016), which also showed above-average reducing potential. As previously presented by [44], antioxidant activity of the *Spirulina* extracts is highly dependent on the extraction solvent. Based on their results, aqueous extracts showed significantly higher antioxidant activity than ethanolic extracts. In one investigation, the extract displayed substantial DPPH radical-scavenging capacity, with IC_50_ around 45.97 µg/mL, outperforming many crude natural extracts in terms of potency [45]. Additionally, ABTS activity was also significant, with an IC_50_ of 31.09 µg/mL, underscoring strong scavenging capabilities against different radical species [45]. Beyond test-tube assays, *Spirulina* aqueous extract demonstrated protective biological effects: in fibroblast (3T3) cell models, it effectively prevented free radical–induced apoptosis, indicating not only in vitro scavenging potential but also functional cellular defense [46]. Comparative analyses further reveal that aqueous extracts often outpace their ethanolic counterparts when yielding enzymatic antioxidative activity, such as catalase, superoxide dismutase (SOD), and glutathione peroxidase (GPx), as well as non-enzymatic phytonutrients like phenols, flavonoids, and tannins [47].

The total protein content (*γ*_TP_) was another major factor influenced by extraction conditions, spanning from 17.531 mg/mL in Exp. 14 to a remarkable 44.566 mg/mL in Exp. 15. Most experiments yielded protein concentrations between 22 and 30 mg/mL, suggesting relatively stable recovery of proteins across different extractions. However, several samples stood out with much higher yields, such as Exp. 3 (34.175 mg/mL), Exp. 22 (31.297 mg/mL), Exp. 23 (32.450 mg/mL), and Exp. 15 (44.566 mg/mL). These results indicate that under certain sonication conditions, protein extraction can be significantly enhanced, likely due to more effective cell wall disruption. Lower protein yields, as observed in Exp. 14 and Exp. 5, may reflect less effective treatment or partial protein degradation. Recent studies demonstrated protein recovery rates of up to 97% when combining UAE with membrane filtration at optimized sonication frequencies and power [48]. Similarly, manothermosonication increased yields to 28.42 g per 100 g dry *Spirulina*, representing a 229% improvement over conventional methods [35]. Other investigations using alkaline solubilization with UAE reported protein yields exceeding 75% while maintaining amino acid integrity [49]. These results demonstrate that *Spirulina platensis* aqueous extracts not only provide an abundant protein source but also benefit from advanced extraction methods that maximize recovery and preserve bioactivity, underscoring their value for food, pharmaceutical, and biotechnological industries.

The C-phycocyanin (CPC) concentration showed the greatest variability among the measured variables. Values ranged from as low as 0.300 mg/mL in Exp. 26 to as high as 5.748 mg/mL in Exp. 16. This wide range illustrates the sensitivity of phycocyanin yield to extraction conditions. Several experiments yielded high CPC concentrations, including Exp. 2 (4.797 mg/mL), Exp. 21 (4.241 mg/mL), and Exp. 25 (3.968 mg/mL), indicating highly favorable extraction conditions for this pigment-protein complex. Conversely, a number of experiments provided very low CPC values below 1 mg/mL, such as Exp. 3, Exp. 4, Exp. 13, and Exp. 26. This inconsistency suggests that while ultrasound-assisted extraction has the potential to efficiently recover phycocyanin, it is also prone to significant variation, possibly due to pigment sensitivity to sonication intensity or differences in stability under the applied conditions. Research has shown yields ranging from less than 1 mg/g to over 40 mg/g of dry biomass, depending on the extraction method. For instance, one study reported a yield of 41.886 mg/g using a simple maceration method with distilled water, while another found a yield of 43.75 mg/g using ultrasonic treatment with glass pearls [50,51,52]. Furthermore, as described in the literature, single-step aqueous extraction methods often yield low-purity extracts. Subsequent purification steps, such as ammonium sulfate precipitation, ion-exchange chromatography, or aqueous two-phase extraction (ATPE), are used to achieve higher purity [53,54,55,56]. For example, some studies have achieved purities of over 4.0 using a combination of extraction and purification techniques [57,58].

**Figure 1:**
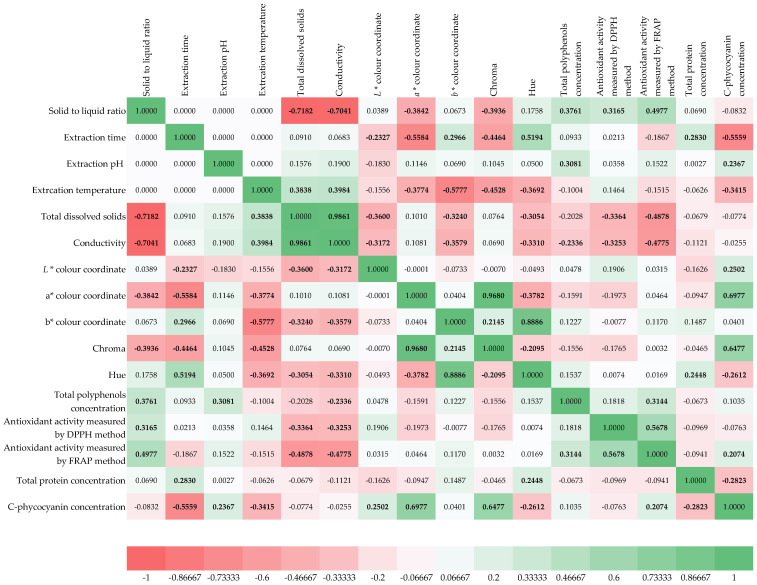
Spearman correlation matrix of extraction conditions and physicochemical properties of prepared *Spirulina platensis* algae extracts. Correlations significant at *p* < 0.05 are marked bold.

The correlation matrix of *Spirulina platensis* extracts provides valuable insights into the relationships among processing conditions and physicochemical properties of the prepared extracts (Figure 1). The color-coded heat map highlights both strong positive and negative correlations, reflecting how extraction variables influence solubility, color parameters, bioactive compounds, and antioxidant activities. Starting with the processing conditions, the solid-to-liquid ratio displayed negative correlations with total dissolved solids (−0.678) and conductivity (−0.704), indicating that higher dilution reduced solubilized compounds and ionic strength in the extracts [59,60]. Extraction temperature correlated positively with both total dissolved solids (0.838) and conductivity (0.839), suggesting that higher temperatures improved cellular disruption and release of soluble material. Extraction pH showed moderate positive associations with protein concentration (0.345) and phycocyanin concentration (0.267), implying that slightly alkaline conditions may favor pigment and protein solubilization [61]. The physicochemical variables were highly interconnected. Total dissolved solids and conductivity were strongly correlated (0.918), confirming that ionic content drives conductivity. Lightness (L*) was negatively related to TDS (−0.315) and conductivity (−0.374), meaning extracts with higher solubilized material appeared darker. The a* coordinate (red-green) correlated positively with total proteins (0.345), suggesting protein-rich extracts shift slightly toward reddish tones. Meanwhile, b* (yellow-blue) correlated strongly with Hue (0.866), reflecting their joint contribution to color characterization. Chroma, representing color intensity, showed a positive correlation with phycocyanin (0.647), confirming that higher pigment concentrations enhance saturation. Among the bioactive compounds, total polyphenol concentration was positively associated with antioxidant capacity by the DPPH method (0.477), aligning with the known radical scavenging role of polyphenols. However, polyphenols showed only a weak correlation with FRAP (0.112), suggesting their reducing capacity may not dominate extract antioxidant behavior [62]. Interestingly, FRAP was more strongly linked to C-phycocyanin (0.422), pointing toward pigments as important contributors to reducing activity. The protein and pigment fractions revealed distinct behaviors. Total proteins correlated positively with extraction pH (0.345) and extraction time (0.248), implying that longer and slightly more alkaline conditions improved protein release. Phycocyanin concentration correlated strongly with Chroma (0.647) and moderately with FRAP activity (0.422), underscoring its dual role as both a color determinant and antioxidant compound. Overall, the correlation matrix highlights that extraction temperature and solid-to-liquid ratio are the most decisive processing variables, directly shaping solubility and conductivity. In turn, these physicochemical properties influence both appearance and biochemical composition. Pigments, especially phycocyanin, emerge as key drivers of extract color and antioxidant potential, while polyphenols primarily contribute to radical scavenging activity measured by DPPH.

### 3.2. Optimization of Extraction Conditions for Biologically Active Molecules from the Blue-Green Alga Spirulina platensis

**Figure 2:**
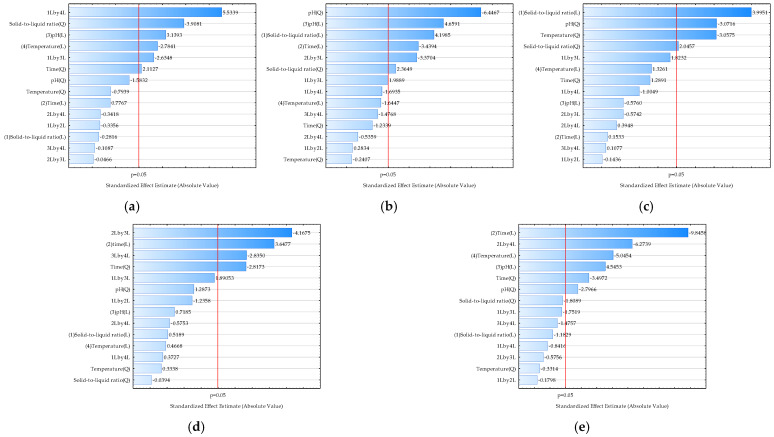
Pareto charts illustrating the effect of extraction conditions on selected properties of *Spirulina platensis* blue-green algae extracts: (**a**) total polyphenol concentration (*R*^2^ = 0.6481, *R*^2^_adj_ = 0.5873, *p* < 0.05); (**b**) antioxidant activity determined by the DPPH method (*R*^2^ = 0.4840, *R*^2^_adj_ = 0.4292, *p* < 0.05); (**c**) antioxidant activity determined by the FRAP method (*R*^2^ = 0.6823, *R*^2^_adj_ = 0.5612, *p* < 0.05); (**d**) total protein concentration (*R*^2^ = 0.4937, *R*^2^_adj_ = 0.4322, *p* < 0.05); (**e**) C-phycocyanin concentration (*R*^2^ = 0.7361, *R*^2^_adj_ = 0.6868, *p* < 0.05). 1—Solid-to-liquid ratio, 2—extraction time, 3—extraction pH, 4—extraction temperature. L-linear term, Q-quadratic term.

**Figure 3:**
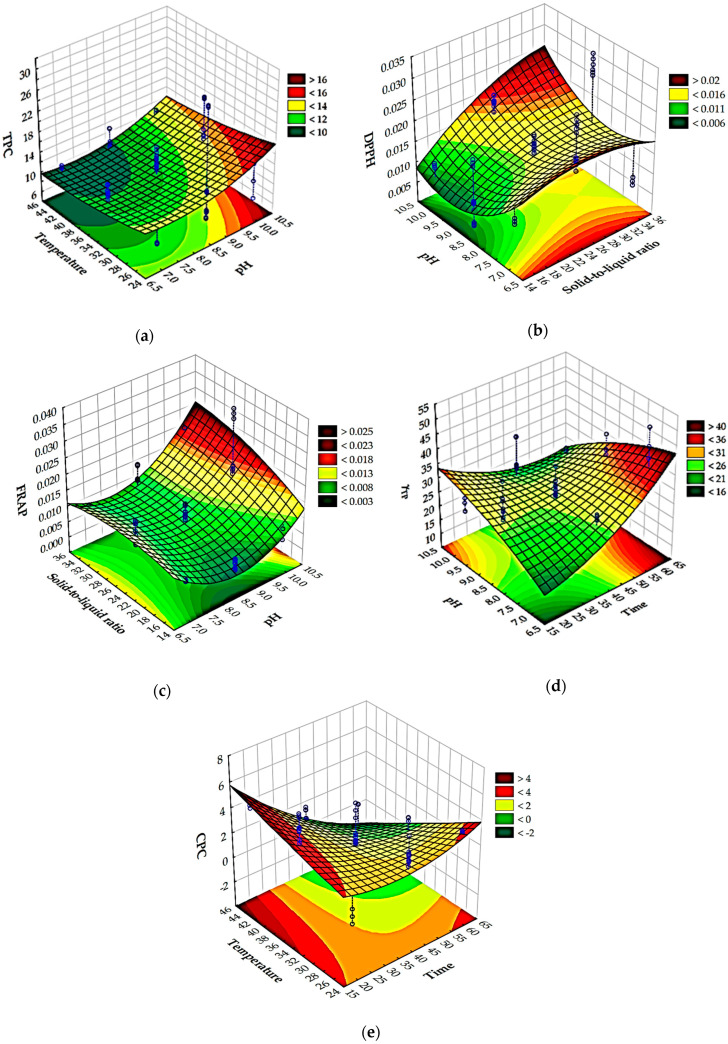
Three-dimensional Response Surface Methodology (RSM) plots for (**a**) total polyphenol concentration, (**b**) antioxidant activity determined by the DPPH method, (**c**) antioxidant activity determined by FRAP method, (**d**) total protein concentration, and (**e**) C-phycocyanin concentration.

Optimizing the extraction of biologically active molecules from *Spirulina platensis* is of paramount importance because it directly influences the yield, purity, and bioactivity of the final product. Many of these compounds, such as the powerful antioxidant C-phycocyanin, are sensitive to environmental factors like temperature and pH. Suboptimal conditions can lead to their degradation, rendering the final product less effective or even useless for commercial applications. A poorly optimized process is also economically unfeasible, as it can result in low yields and a high cost of production. Therefore, it is essential to find the ideal “sweet spot” where extraction is most efficient without compromising the integrity of the desired molecules [63]. In this work, optimization using a desirability function was applied to simultaneously estimate the optimal extraction conditions to achieve a maximum total polyphenol concentration, antioxidant activity determined by the DPPH method, antioxidant activity determined by the FRAP method, total protein concentration, and C-phycocyanin concentration based on the Box-Benkhen design of the experiments. The influences of the extraction conditions on the analyzed model variables described by RSM equations were analyzed using Pareto diagrams (Figure 2) and 3D plots (Figure 3). The Pareto chart visually ranks the influence of each extraction condition (like temperature, pH, or time) on the analyzed properties, and this helps to identify the most significant factors, allowing us to focus on controlling the “vital few” variables that have the greatest impact on the extract’s quality. Furthermore, 3D RSM plots are used to visualize the relationship between two independent variables and a single dependent variable (the response). They are a key tool in process optimization because they help to visually identify the ideal combination of factors to achieve a maximum, minimum, or target response. Based on the provided Pareto chart and RSM plots (Figure 2a and Figure 3a), the most significant factor affecting the total polyphenol concentration is the solid-to-liquid ratio (Q), followed by pH (L), temperature (L), and time (L). In the case of antioxidant activity determined by the DPPH method (Figure 2b and Figure 3b), solid-to-liquid ratio (Q) was again shown to be the most important variable, while in the case of antioxidant activity determined by the FRAP method, pH (Q) was the most important variable (Figure 2c and Figure 3c). Results also showed that time has the most effect on the total protein concentration (Figure 2d and Figure 3d) and on the C-phycocyanin concentration (Figure 2e and Figure 3e).

**Figure 4:**
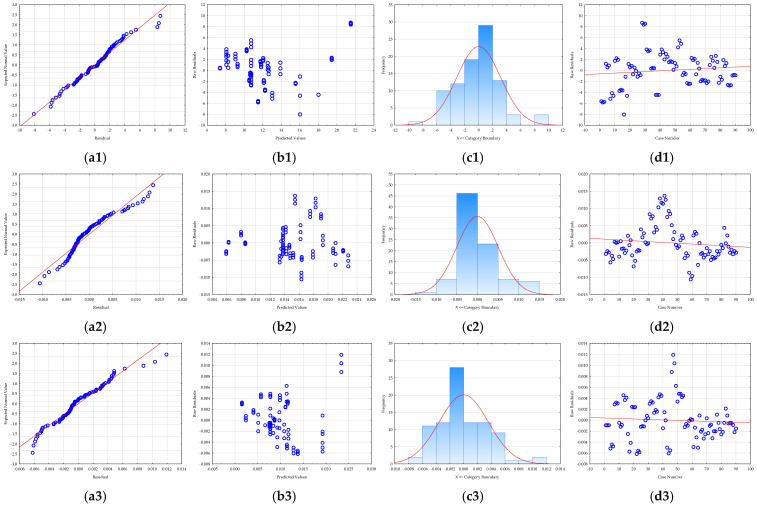

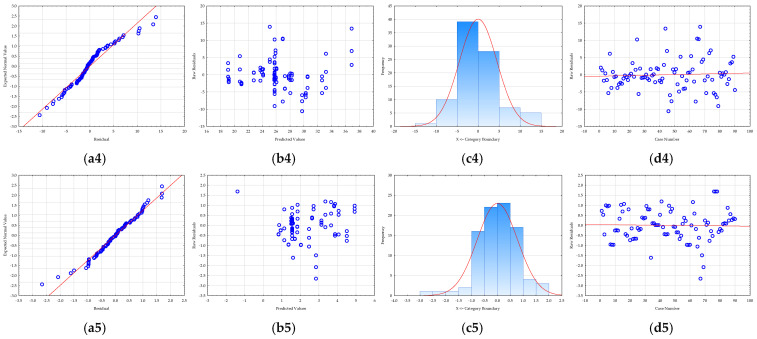
RSM model residual analysis: (**a**) normality plot, (**b**) dependence between model predicted values and residuals, (**c**) histogram of residuals, and (**d**) dependence between experiment number and residual value for (1) total polyphenol concentration, (2) antioxidant activity determined by the DPPH method, (3) antioxidant activity determined by FRAP method, (4) total protein concentration, and (5) C-phycocyanin concentration.

Prior to the optimization step, it is crucial to evaluate the adequacy of the developed RSM models [64]. Therefore, residual analysis was used (Figure 4). A residual is the difference between an observed, experimental value and the value predicted by the model. By analyzing these residuals, researchers can assess whether the assumptions of the model are being met. All models showed that the residuals were normally distributed, an essential assumption confirmed in two ways: first, the residuals were closely aligned along a straight line in normal probability plots (Figure 4), and second, the histograms (Figure 4) of the residual distribution exhibited a classic bell-shaped curve with minimal skewness. Furthermore, the analysis confirmed that the residuals were randomly distributed (Figure 4), which is crucial for a robust model. Plots of residuals against the predicted values showed no discernible patterns (Figure 4), indicating that the models accurately captured the relationships between the variables and that no systematic bias was present. A plot of residuals versus the experimental run order (Figure 4) confirmed that the randomization of the experiments was successful and that the sequence of tests did not influence the results. According to Salehi et al. [65], this implies that the level of randomization was adequate and that the order of testing did not influence the data. Overall, the residual analysis confirmed that the developed RSM models are statistically sound, with no evidence of model inadequacy or non-random errors, ensuring the reliability of the optimization results.

In essence, a proper residual analysis provides confidence that the model is a good fit for the data. If the residuals are well-behaved and meet the necessary assumptions, the model’s predictions for process optimization are considered reliable. Without this crucial step, the optimization results derived from the RSM could be misleading, potentially leading to incorrect conclusions and an unoptimized process in practice.

**Figure 5:**
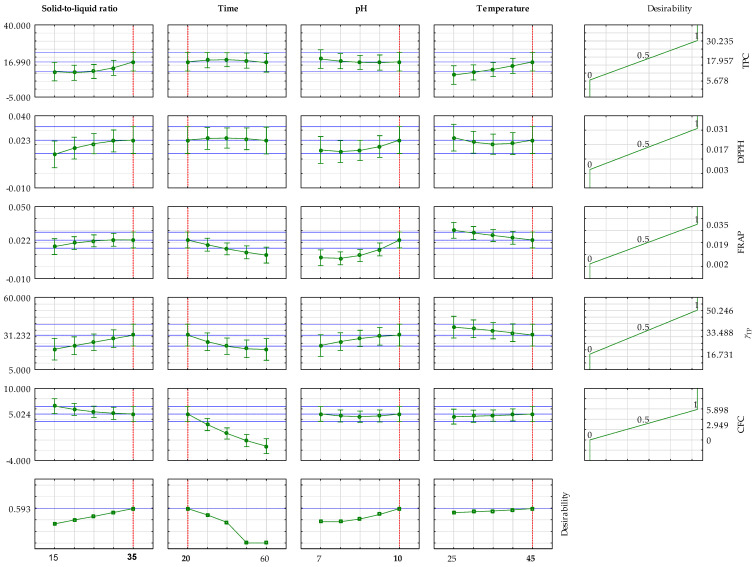
Assessment of the optimal conditions for achieving the maximum concentration of total polyphenols, antioxidant activity determined by the DPPH method, antioxidant activity determined by the FRAP method, total protein concentration, and C-phycocyanin concentration.

Figure 5 presents an assessment of the optimal conditions for achieving the maximum concentration of total polyphenols, antioxidant activity (DPPH and FRAP methods), total protein concentration, and C-phycocyanin concentration. Instead of optimizing each property individually, the desirability function was employed to combine all these objectives into a single goal. This approach is crucial because conditions that might maximize one desired property could be detrimental to another. The desirability function, therefore, identifies a compromise or an optimal extraction condition that yields the best overall result across all measured outcomes. It converts each response into a dimensionless “desirability” score (from 0 to 1), where 1 is perfect and 0 means a goal was not met, then calculates a composite desirability. The plots show the effect of each variable (solid-to-liquid ratio, time, pH, and temperature) on each response, with the vertical red dashed lines indicating the derived optimal settings for each factor. The final column illustrates the desirability for each response, culminating in an overall desirability of approximately 0.6, signifying a good, balanced compromise across all the targeted properties. As presented, the estimated optimal conditions were as follows: S/L = 35 g/L, *t* = 20 min, pH = 10, and *T* = 45 °C.

The independent validation of the estimated optimal conditions was performed. The results are presented in Table 3. This step is essential because while a statistical model can predict an optimal outcome, its real-world effectiveness must be confirmed through an independent, real-world experiment. The comparison between the RSM-predicted values and the values obtained from the validation experiment under the estimated optimal conditions demonstrates the model’s accuracy and reliability. Across all five measured variables—total polyphenols (TPC), antioxidant activities (DPPH and FRAP), total protein content (g), and C-phycocyanin concentration (CPC)—there is a remarkable concordance between the predicted and experimental results. For instance, the RSM model predicted a DPPH value of 0.0240 mmol_TE_/g_dw_, which is nearly identical to the experimental value of 0.0242 ± 0.003 mmol_TE_/g_dw_. Similarly, the predicted FRAP value of 0.0225 mmol_FeSO_4_·7H_2_O_/g_dw_ is very close to the experimental value of 0.0219 ± 0.002 mmol_FeSO_4_·7H_2_O_/g_dw_. This high degree of consistency validates the model’s ability to accurately forecast the outcome of the extraction process. The minor deviations observed, such as the predicted TPC of 17.3103 mg_GAE_/g_dw_ versus the experimental 16.1115 ± 0.888 mg_GAE_/g_dw_, are both expected and acceptable. They are well within the typical range of experimental error inherent in biological systems and laboratory procedures. These small differences do not undermine the model’s effectiveness; instead, they highlight the robustness of the methodology. The presence of small standard deviations in the experimental values also indicates that the independent validation runs were reproducible, adding another layer of confidence in the results. In essence, the validation data presented in the table confirms that the optimal conditions identified through the Box–Behnken design and desirability function are not just theoretical but are practically achievable. This result is of great importance for future applications, as it proves that the developed protocol is a reliable and scalable method for efficiently extracting valuable bioactive compounds from *Spirulina platensis*. The successful validation bridges the gap between statistical modeling and practical application, providing a solid foundation for further research or potential industrial-scale production.

### 3.3. NIR Spectra of Blue-Green Algae Spirulina platensis Extracts and Artificial Neural Network Models for Predicting Physicochemical Properties of Spirulina platensis Extracts Based on NIR Spectra

**Figure 6:**
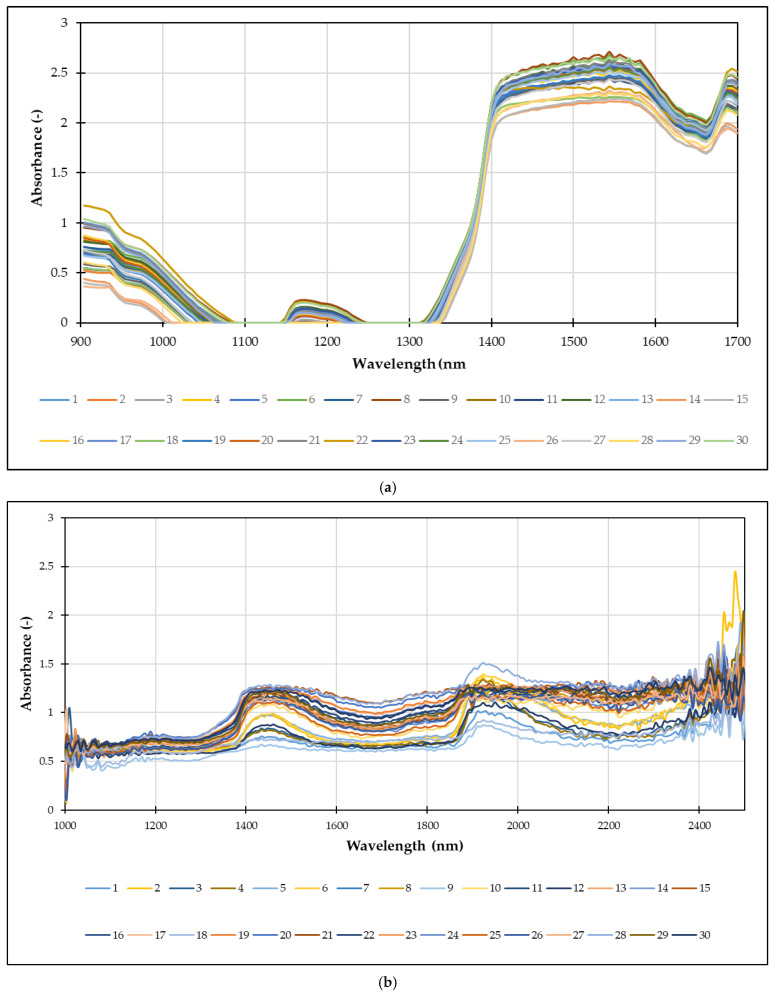
Average raw NIR spectra of *Spirulina* extracts were recorded using: (**a**) semi-process NIR spectrometer and (**b**) benchtop NIR spectrometer.

NIR spectroscopy measures the absorption of light in the Near-Infrared region (780–2500 nm). The unique absorption patterns, or spectra, of *Spirulina* extracts are directly related to the overtones and combination bands of molecular bonds such as O-H, C-H, and N-H. These bonds are found in key components like water, proteins, carbohydrates, and lipids. Every extract, based on its specific composition, will produce a unique NIR spectrum, serving as a kind of “fingerprint” of its physicochemical properties. This allows NIR spectroscopy to be used for the following: identifying and quantifying the concentration of various compounds, such as total polyphenols, total proteins, and C-phycocyanin [66], and assessing the overall antioxidant activity of the extract and monitoring the extraction process in real-time [67]. In this work, NIR spectra of *Spirulina* extracts were recorded using two NIR instruments: semi-process NIR spectrometer and a benchtop NIR spectrometer. Average raw spectra are given in Figure 6.

For semi-process NIR spectrometer (Figure 6a) the graph plots absorbance on the *y*-axis against wavelength (nm) on the *x*-axis, covering a range from 900 nm to 1700 nm. The spectra show distinct regions of high absorbance, particularly in the range of 900–1000 nm and from approximately 1350 nm onwards. These absorption bands correspond to the overtones and combination bands of various chemical bonds (e.g., O-H, C-H, N-H) present in the extracts. In case of benchtop NIR spectrometer (Figure 6b) the raw spectra appear complex and noisy, therefore chemometrics approach, such as PCA with an Artificial Neural Network, can be used to extract meaningful quantitative information from these unique “fingerprints.”

**Figure 7:**
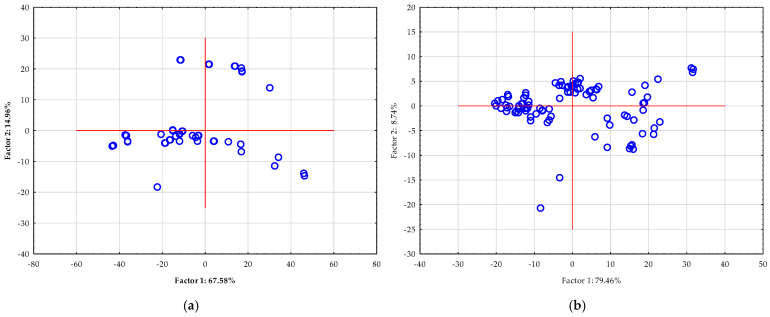
PCA of raw NIR spectra of *Spirulina* extracts were recorded using: (**a**) semi-process NIR spectrometer and (**b**) benchtop NIR spectrometer.

NIR spectra contain a wealth of information, but they are often highly complex due to a large number of correlated variables (wavelengths). This complexity can make direct interpretation difficult. PCA addresses this challenge by reducing the dimensionality of the data. It transforms the original set of variables into a new, smaller set of uncorrelated variables called principal components (PCs). These PCs capture the maximum variance in the data, effectively distilling the most important information into a manageable form. Therefore, by using PCA, researchers can perform the following: (i) visualize data clusters: the scatter plot shows how the different samples relate to each other. Samples with similar compositions will group together, while those that are distinct will appear as separate clusters; (ii) identify outliers: data points that fall far from the main cluster can be easily identified as outliers, which may indicate an issue with the sample or the measurement process; and (iii) remove noise: PCA can help to filter out irrelevant noise from the spectra, focusing only on the chemical information that is most relevant to the study [68]. Figure 7 shows the results of a PCA of NIR spectra from *Spirulina extracts*. In the case of semi-process NIR, first two principal components, which account for a significant portion of the total variance in the dataset (67.58% and 14.96%, respectively, for a cumulative 82.54%). In the case of benchtop NIR, first two principal components account for a significant portion of the total variance in the dataset (79.46% and 8.74%, respectively, for a cumulative 86.20%). The scatter of the data points for both NIR instruments suggests some variability in the composition of the extracts. Most of the data points are clustered around the center, indicating similar overall chemical profiles, but some points extend into the quadrants, suggesting minor differences in their composition. This visual representation is critical for understanding the overall variability within the set of extracts and serves as a valuable preliminary step before building predictive models like those using Artificial Neural Networks (ANN).

The results from PCA were further used to develop an efficient ANN predictive model to describe selected physicochemical properties of *Spirulina* extract based on the NIR spectra recorded using a semi-process NIR spectrometer and a benchtop NIR spectrometer. The ANN model’s input was the first five PCs from PCA, contributing to 99% of data variability. ANN modeling using NIR spectra is highly important for several reasons. Firstly, it offers a rapid and non-destructive alternative to conventional, time-consuming laboratory analyses. Instead of complex chemical procedures, a simple scan of the sample provides a wealth of information. Secondly, NIR spectra are complex and contain overlapping signals, which traditional statistical methods struggle to interpret effectively. ANNs, as a powerful form of machine learning, excel at identifying the non-linear, intricate relationships between the spectral data and the corresponding properties, acting as a sophisticated pattern-recognition tool. This allows for accurate prediction of key quality indicators like total polyphenols and antioxidant activity, which are crucial for product quality control. Table 4 reveals that Multilayer Perceptron (MLP) models were used for all predictions. The architecture, denoted as MLP X-Y-1, indicates the number of input neurons (X), hidden neurons (Y), and one output neuron for the predicted property. The results show that the models trained on the semi-processed NIR spectrometer data generally performed better, as indicated by higher *R*^2^ values and lower RMSE values across the board. For instance, a model for L* based on NIR spectra gathered using a semi-process NIR instrument showed a high validation *R*^2^ of 0.9105, indicating that over 91% of the variance was explained by the model. The benchtop models, while still useful, showed a wider drop in performance from the training to the validation set. This suggests that the semi-process spectrometer may be better suited for this application. The specific activation functions used, such as Tanh, Logistic, Exponential, and Identity, were selected to optimize the learning process for each specific property. Ultimately, this detailed analysis confirms the viability and effectiveness of using ANN models with NIR spectroscopy for a fast and reliable assessment of *Spirulina extract* quality.

By now numerous studies have successfully utilized NIR spectroscopy to predict key properties of *Spirulina* extracts, demonstrating its potential as a rapid and cost-effective analytical tool. The most significant results show that NIR models, particularly when combined with advanced chemometric techniques like ANN, can accurately predict protein content, total polyphenol concentration, and antioxidant activity (measured by assays like DPPH and FRAP) with high coefficients of determination [66]. This is particularly valuable for both quality control during extraction and for monitoring the growth of *Spirulina* biomass in real-time, as it bypasses the need for time-consuming and labor-intensive traditional laboratory methods [67]. For instance, studies have successfully used portable NIR spectrometers to monitor extraction yield and protein concentration, proving the feasibility of on-site analysis. This non-destructive approach also means the sample remains intact, allowing for further use. However, this approach is not without its limitations. A primary challenge is the complexity of the NIR spectrum itself [69,70,71]. Unlike mid-infrared spectroscopy, the NIR region consists of broad, overlapping overtone and combination bands of various chemical bonds (O-H, C-H, N-H). This makes direct interpretation difficult and necessitates sophisticated multivariate statistical models. The presence of water is a major interferent, as its strong absorbance bands can overlap with those of other key compounds, especially in aqueous extracts. This can make it difficult to accurately quantify certain components without specific data preprocessing. Furthermore, the model’s robustness is highly dependent on the calibration dataset. A model developed for one set of cultivation conditions or extraction methods may not perform well on samples with different characteristics, highlighting the need for a diverse and representative sample set.

Predictive performance of developed ANN models was evaluated on the independent dataset, which is a critical step for validating their real-world applicability. Table 5 presents key statistical metrics for models built using NIR spectra from both a semi-process and a benchtop spectrometer. The validation was performed on an independent dataset, and the results are evaluated using key statistical metrics such as *R*^2^_pred_, RMSEP, RPD, and RER. The RPD is a particularly important metric, as it provides a clear indication of a model’s predictive capability, with values greater than 3 indicating excellent performance, between 2 and 3 indicating good performance, and less than 2 indicating suitability for screening or poor performance. The results show a clear difference in the models’ performance based on the instrument used. The semi-process NIR spectrometer generally yielded superior models. Its models for L* and TDS were particularly strong, with RPD values of 3.9064 and 2.8351, respectively. This indicates that these models have excellent to good predictive ability. Other models from the semi-process spectrometer, such as those for TPC, DPPH, FRAP, and *γ*_TP_, had RPDs ranging from 1.6084 to 2.1752, making them suitable for preliminary screening. In contrast, the benchtop NIR spectrometer produced models that were less robust. While its models for TDS and L* were still in the good predictive range with RPDs of 2.3080 and 2.0499, the performance for all other variables was notably weaker. The models for TPC, FRAP, and *γ*_TP_ fell into the lower end of the screening category, and the models for DPPH and CPC had RPDs of 1.4079 and 1.3491, respectively, indicating poor predictive power. Overall, the comparison demonstrates that the semi-process NIR spectrometer provided higher quality spectral data, which translated into more accurate and reliable predictive models for the physicochemical properties of *Spirulina* extracts. This could be explained by the instrument resolution; e.g., a semi-process NIR spectrometer has higher resolution and consequently generates higher-quality spectral data. NIR instruments with high resolution can separate very narrow absorption bands, which is crucial for identifying and quantifying specific chemical components [72]. This is because the spectral “fingerprints” of different molecules can overlap. Low-resolution instruments, on the other hand, will average these closely spaced bands together, resulting in a single, broad peak [73]. This can lead to loss of critical information, making it difficult to distinguish between different compounds or accurately measure their concentrations. This makes it a better choice for this specific application.

## 4. Conclusions

This research successfully optimized the UAE of bioactive compounds from *Spirulina platensis* and developed a robust, rapid analytical method using NIR spectroscopy coupled with ANN models. The study revealed that UAE is a highly effective method for recovering valuable compounds, including polyphenols, proteins, and C-phycocyanin, but its efficiency is critically dependent on process parameters. Through RSM and a desirability function, we identified the optimal extraction conditions—a solid-to-liquid ratio of 35 g/L, a time of 20 min, a pH of 10, and a temperature of 45 °C—that yielded the best overall results. The subsequent validation of these conditions confirmed the model’s accuracy, with a strong concordance between predicted and experimental values. This successful validation is a key result, as it proves the developed protocol is not just a theoretical model but a practical and reproducible method for producing high-quality *Spirulina* extracts on a larger scale.

The most significant achievement of this work is the development of predictive NIR-ANN models that can accurately assess extract quality in a non-destructive and real-time manner. We demonstrated that the models built on data from a semi-process NIR spectrometer were superior to those from a benchtop instrument, with excellent predictive performance for properties like lightness (L*) and total dissolved solids (TDS) and good performance for other key variables. These results indicate the crucial role of instrument resolution in the quality of spectral data and, consequently, the accuracy of predictive models. By establishing a direct link between the spectral “fingerprint” of the extract and its chemical composition, this research provides a powerful, automated tool for quality control that bypasses the need for traditional, time-consuming laboratory analyses. The successful integration of an optimized extraction method with an intelligent analytical tool represents a comprehensive and powerful approach to modern biotechnology.

Based on our results, several exciting avenues for future research and industrial application emerge. The validated UAE protocol can serve as a foundation for scaling up to industrial extraction systems, supporting cost-effective production of *Spirulina* extracts for functional foods, pharmaceuticals, and cosmetics. Its reproducibility and efficiency make it highly adaptable to large-scale biorefinery settings, where throughput and consistency are essential. In parallel, the most promising future direction lies in the deployment of NIR-ANN models for smart extraction monitoring, enabling real-time, in-line control of industrial processes. By integrating these models into extraction lines, manufacturers can implement automated, self-optimizing systems that adjust process parameters on the fly to maximize yield, ensure consistent quality, and minimize waste.

Future research should explore the long-term stability and bioavailability of the extracted compounds under optimal conditions to guarantee product efficacy and shelf life. In addition, expanding the calibration dataset to include diverse *Spirulina* strains, cultivation conditions, and solvent systems would enhance the robustness and generalizability of the predictive models. Ultimately, the methodology presented here not only lays the groundwork for sustainable industrial production of high-value *Spirulina* extracts but also paves the way for its application to other microalgae and plant systems, advancing the broader field of rapid, non-destructive, and intelligent natural product extraction.

## Figures and Tables

**Table 1:** Experimental conditions for the preparation of *Spirulina platensis* extracts.

Exp.	S/L (g/L)	pH	*T* (°C)	*t* (min)
1.	15	8.5	35	20
2.	35	8.5	35	20
3.	15	8.5	35	60
4.	35	8.5	35	60
5.	25	7	25	40
6.	25	10	25	40
7.	25	7	45	40
8.	25	10	45	40
9.	25	8.5	35	40
10.	15	8.5	25	40
11.	35	8.5	25	40
12.	15	8.5	45	40
13.	35	8.5	45	40
14.	25	7	35	20
15.	25	7	35	60
16.	25	10	35	20
17.	25	10	35	60
18.	25	8.5	35	40
19.	15	7	35	40
20.	35	7	35	40
21.	15	10	35	40
22.	35	10	35	40
23.	25	8.5	25	20
24.	25	8.5	25	60
25.	25	8.5	45	20
26.	25	8.5	45	60
27.	25	8.5	35	40
28.	25	8.5	35	40
29.	25	8.5	35	40
30.	25	8.5	35	40

**Table 2:** Chemical properties of *Spirulina platensis* blue-green algae extracts prepared by ultrasound-assisted extraction. (*γ*_TP_—total protein concentration; CPC—C-phycocyanin concentration; TPC—total polyphenol concentration; DPPH—antioxidant activity measured by the DPPH method; FRAP—antioxidant activity measured by the FRAP method).

Exp.	TPC (mg_GAE_/g_dw_)	DPPH (mmol_TE_/g_dw_)	FRAP (mmol_FeSO_4_·7H_2_O_/g_dw_)	*γ*_TP_ (mg/mL)	CPC (mg/mL)
1	5.789 ± 0.156	0.003 ± 0.001	0.006 ± 0.000	24.261 ± 2.007	4.334 ± 0.632
2	12.733 ± 0.487	0.009 ± 0.001	0.007 ± 0.001	25.922 ± 2.916	4.797 ± 0.022
3	8.407 ± 0.730	0.006 ± 0.000	0.005 ± 0.000	34.175 ± 4.961	0.400 ± 0.008
4	14.024 ± 0.365	0.012 ± 0.003	0.007 ± 0.001	28.872 ± 1.910	0.686 ± 0.009
5	8.603 ± 0.174	0.018 ± 0.001	0.014 ± 0.001	18.466 ± 0.225	2.529 ± 0.351
6	11.430 ± 4.867	0.020 ± 0.002	0.015 ± 0.002	27.208 ± 0.441	3.903 ± 0.887
7	10.140 ± 0.435	0.018 ± 0.002	0.013 ± 0.000	27.759 ± 0.936	1.113 ± 0.779
8	12.537 ± 0.869	0.020 ± 0.000	0.008 ± 0.001	20.523 ± 1.968	1.035 ± 0.008
9	9.894 ± 0.435	0.017 ± 0.002	0.007 ± 0.000	27.967 ± 7.904	1.368 ± 0.059
10	30.051 ± 0.261	0.008 ± 0.000	0.003 ± 0.001	25.000 ± 0.503	3.001 ± 0.026
11	13.938 ± 0.243	0.027 ± 0.001	0.015 ± 0.000	24.880 ± 0.793	3.565 ± 0.094
12	7.817 ± 0.104	0.018 ± 0.001	0.006 ± 0.000	24.647 ± 0.979	1.141 ± 0.988
13	13.594 ± 0.000	0.030 ± 0.002	0.010 ± 0.000	26.519 ± 2.264	0.877 ± 0.003
14	11.430 ± 0.695	0.028 ± 0.002	0.010 ± 0.002	17.531 ± 0.547	3.887 ± 0.674
15	11.553 ± 0.695	0.026 ± 0.001	0.007 ± 0.001	44.566 ± 5.330	0.388 ± 0.013
16	13.335 ± 0.087	0.019 ±0.003	0.034 ± 0.002	21.680 ± 2.343	5.748 ± 0.150
17	13.274 ± 0.869	0.014 ± 0.001	0.016 ± 0.002	25.229 ± 0.572	1.682 ± 0.163
18	15.609 ± 0.869	0.015 ± 0.001	0.012 ± 0.001	24.987 ± 5.314	1.041 ± 0.169
19	10.029 ± 0.313	0.011 ± 0.001	0.007 ± 0.000	24.430 ± 2.092	1.734 ± 0.150
20	13.163 ± 0.122	0.007 ± 0.001	0.009 ± 0.000	22.634 ± 1.308	1.063 ± 0.005
21	21.570 ± 0.261	0.011 ± 0.001	0.006 ± 0.002	22.437 ± 3.671	4.241 ± 0.577
22	14.282 ± 1.460	0.018 ± 0.005	0.017 ± 0.004	31.297 ± 9.507	1.847 ± 0.438
23	8.726 ± 0.174	0.012 ± 0.001	0.009 ± 0.001	32.450 ± 5.702	0.765 ± 0.022
24	10.078 ± 0.348	0.012 ± 0.001	0.007 ± 0.002	30.150 ± 2.564	3.268 ± 0.142
25	9.833 ± 1.043	0.012 ± 0.001	0.009 ± 0.002	29.900 ± 4.699	3.968 ± 0.345
26	9.833 ± 1.564	0.015 ± 0.001	0.005 ± 0.002	24.358 ± 0.613	0.300 ± 0.004
27	9.034 ± 0.782	0.012 ± 0.001	0.006 ± 0.001	22.775 ± 5.267	1.294 ± 0.075
28	12.106 ± 0.782	0.016 ± 0.003	0.009 ± 0.002	26.170 ± 0.321	1.641 ± 0.015
29	8.050 ± 0.087	0.012 ± 0.001	0.007 ± 0.000	25.598 ± 3.105	2.110 ± 0.322
30	9.894 ± 0.087	0.011 ± 0.001	0.006 ± 0.001	27.264 ± 5.200	1.862 ± 0.040

**Table 3:** Comparison of predicted (RSM) and experimental values from the independent validation experiment under estimated optimal conditions.

Variable	RSM Predicted Value	Validation Experiment
TPC (mg_GAE_/g_dw_)	17.3103	16.1115 ± 0.888
DPPH (mmol_TE_/g_dw_)	0.0240	0.0242 ± 0.003
FRAP (mmol_FeSO_4_·7H_2_O_/g_dw_)	0.0225	0.0219 ± 0.002
*γ*_TP_ (mg/mL)	24.2727	26.8771 ± 2.037
CPC (mg/mL)	5.1829	5.2008 ± 0.335

**Table 4:** Architecture of ANN model develop to predict selected physicochemical properties of *Spirulina* extract’s based on the NIR spectra recorded using semi-process NIR spectrometer and benchtop NIR spectrometer.

NIR	Output Variable	ANN Model	*R*^2^_train_/RMSE_train_	*R*^2^_test_/ RMSE_test_	*R*^2^_validation_/ RMSE_validation_	Hidden Activation	Output Activation
Semi-process NIR spectrometer	TDS	MLP 5-3-1	0.9285 325.3038	0.9463 332.5314	0.9377 466.1069	Tanh	Exponential
	L*	MLP 5-5-1	0.9669 0.0626	0.9934 0.1333	0.9105 0.2402	Logistic	Identity
	TPC	MLP 5-8-1	0.9240 2.1196	0.8899 2.6527	0.8685 2.8634	Logistic	Exponential
	DPPH	MLP 5-5-1	0.9426 0.0001	0.7858 0.0001	0.7679 0.0001	Tanh	Exponential
	FRAP	MLP 5-3-1	0.9277 0.0001	0.8791 0.0001	0.8477 0.0001	Tanh	Identity
	*γ*_TP_	MLP 5-10-1	0.9032 2.6459	0.9009 5.1279	0.7942 10.4731	Tanh	Exponential
	CPC	MLP 5-10-1	0.9472 0.3884	0.7919 0.4029	0.7766 0.5166	Tanh	Identity
Benchtop NIR spectrometer	TDS	MLP 5-4-1	0.9743 285.9742	0.8362 584.2002	0.8535 761.5806	Tanh	Tanh
	L*	MLP 5-11-1	0.8298 2.9197	0.7593 5.2125	0.7624 8.7863	Logistic	Logistic
	TPC	MLP 5-11-1	0.8955 0.0001	0.8851 0.0001	0.7333 0.0001	Tanh	Tanh
	DPPH	MLP 5-8-1	0.8901 0.0001	0.7897 0.0001	0.5755 0.0001	Tanh	Exponential
	FRAP	MLP 5-6-1	0.8743 0.0003	0.7846 0.0009	0.5985 0.00012	Logistic	Identity
	*γ*_TP_	MLP 5-8-1	0.8742 6.6599	0.7845 7.6108	0.5913 12.5533	Tanh	Logistic
	CPC	MLP 5-8-1	0.9363 0.2196	0.7285 0.5854	0.6913 0.6052	Tanh	Logistic

**Table 5:** Predictive performance of developed ANN models based on the NIR spectra recorded using semi-process NIR spectrometer and benchtop NIR spectrometer.

NIR	Output Variable	*R* ^2^_pred_	*R* ^2^_pred,adj_	RMSEP	SEP	RPD	RER
Semi-process NIR spectrometer	TDS	0.8780	0.8503	61.4259	11.8214	2.8351	13.3494
	L*	0.9440	0.9313	0.6401	0.1182	3.9064	21.1236
	TPC	0.8121	0.7694	2.0706	0.3985	2.1752	11.8596
	DPPH	0.7320	0.6711	0.0038	0.0007	1.9063	6.9911
	FRAP	0.6866	0.6154	0.0036	0.0007	1.6084	8.4019
	*γ*_TP_	0.6704	0.5955	3.5027	0.6741	1.6643	6.4703
	CPC	0.6676	0.5921	1.0008	0.1926	1.7296	5.7099
Benchtop NIR spectrometer	TDS	0.8060	0.7620	109.6030	21.0931	2.3080	8.6768
	L*	0.7565	0.7011	1.3358	0.2571	2.0499	10.9971
	TPC	0.6693	0.5942	2.8229	0.5433	1.6724	7.9024
	DPPH	0.4794	0.3610	0.0041	0.0008	1.4079	5.2696
	FRAP	0.6634	0.5869	0.0044	0.0008	1.5026	7.4504
	*γ*_TP_	0.5897	0.4965	3.2396	0.6235	1.4901	6.7187
	CPC	0.4417	0.3148	1.1123	0.2161	1.3491	4.9848

## Data Availability

The data are available on request.

## Supplementary Materials

The following supporting information can be downloaded at: https://www.mdpi.com/article/10.3390/foods14193358/s1, Table S1: Physical properties of *Spirulina platensis* blue-green algae extracts prepared by ultrasound-assisted extraction. (TDS-total dissolved solids; G-conductivity; *L**, *a**, *b** coordinates of color; Hue- pure spectrum of colors; Chroma-color saturation).
