# Supplementary material for: Ultrasound-Assisted Extraction of Bioactives from Spirulina platensis: Optimization and Prediction of Their Properties Using Near-Infrared Spectroscopy Coupled with Artificial Neural Network Modeling
Source: Foods. 2025 Sep 28;14(19):3358. doi: 10.3390/foods14193358 (PMC12523758; doi:10.3390/foods14193358)
Supplement: Supplementary file 1 [file foods-14-03358-s001.zip › foods-3875727-supplementary.pdf]

Supplementary Table S1. Physical properties of *Spirulina platensis* blue-green algae extracts prepared by ultrasound-assisted extraction. (TDS-total dissolved solids; G-conductivity; *L**, *a**, *b** coordinates of color; Hue- pure spectrum of colors; Chroma-color saturation)

Exp.	TDS (mg/L)	G (μS/cm)	*L**	*a**	*b**	Hue	Chroma
1	1464 ± 14.1	2995 ± 21.2	41.66 ± 0.01	2.41 ± 0.01	0.31 ± 0.007	7.23 ± 0.170	2.43 ± 0.01
2	991 ± 1.4	1974.5 ± 3.5	42.81 ± 0.00	1.45 ± 0.00	0.07 ± 0.007	2.52 ± 0.156	1.46 ± 0.01
3	1594.5 ± 6.4	3225 ± 7.1	41.55 ± 0.00	0.56 ± 0.01	0.41 ± 0.014	36.33 ± 1.245	0.70 ± 0.01
4	1026 ± 1.4	2040 ± 0.0	41.66 ± 0.00	0.19 ± 0.01	0.47 ± 0.007	68.25 ± 1.110	0.50 ± 0.01
5	927.5 ± 2.1	1840.5 ± 0.7	54.93 ± 1.22	1.44 ± 0.06	0.37 ± 0.127	14.35 ± 5.558	1.48 ± 0.03
6	937.5 ± 7.8	1927 ± 5.7	42.36 ± 0.01	1.81 ± 0.01	1.04 ± 0.014	29.77 ± 0.453	2.09 ± 0.00
7	1088.5 ± 12.0	2075 ± 49.5	42.4 ± 0.00	0.79 ± 0.00	0.21 ± 0.007	14.52 ± 0.728	0.82 ± 0.00
8	1148.5 ± 9.2	2335 ± 7.1	42.25 ± 0.01	0.60 ± 0.01	0.24 ± 0.000	21.64 ± 0.007	0.65 ± 0.01
9	1202.5 ± 6.4	2430 ± 0.0	42.13 ± 0.00	0.94 ± 0.01	0.06 ± 0.000	3.49 ± 0.035	0.94 ± 0.01
10	1171 ± 2.8	2280 ± 28.3	42.61 ± 0.05	1.67 ± 0.06	1.13 ± 0.014	34.19 ± 0.608	2.02 ± 0.06
11	654 ± 17.0	1348.5 ± 17.7	42.08 ± 0.01	2.16 ± 0.01	1.16 ± 0.007	28.19 ± 0.078	2.44 ± 0.01
12	1541.5 ± 10.6	3060 ± 99.0	41.82 ± 0.00	1.73 ± 0.01	0.07 ± 0.007	2.31 ± 0.255	1.73 ± 0.01
13	956 ± 2.8	1898.5 ± 6.4	41.55 ± 0.01	0.88 ± 0.01	0.03 ± 0.000	2.12 ± 0.212	0.88 ± 0.01
14	1126 ± 8.5	2295 ± 7.1	43.16 ± 0.01	1.29 ± 0.01	0.39 ± 0.007	17.08 ± 0.354	1.36 ± 0.01
15	1178 ± 5.7	2300 ± 28.3	41.57 ± 0.00	0.66 ± 0.01	0.59 ± 0.007	41.67 ± 0.544	0.88 ± 0.01
16	1216 ± 7.1	2465 ± 7.1	41.48 ± 0.01	1.80 ± 0.00	0.21 ± 0.021	6.375 ± 0.629	1.81 ± 0.00
17	1227 ± 1.4	2430 ± 14.1	41.3 0± 0.00	0.97 ± 0.01	0.45 ± 0.007	24.79 ± 0.255	1.07 ± 0.01
18	1297 ± 5.7	2565 ± 7.1	42.03 ± 0.00	1.24 ± 0.00	0.06 ± 0.014	2.71 ± 0.410	1.24 ± 0.00
19	1489 ± 5.7	3000 ± 0.0	41.35 ± 0.01	1.83 ± 0.00	0.08 ± 0.000	2.39 ± 0.057	1.83 ± 0.00
20	963 ± 0.0	1932 ± 11.3	41.54 ± 0.00	0.73 ± 0.00	0.35 ± 0.070	25.38 ± 0.962	0.81 ± 0.00
21	1600.5 ± 10.6	3230 ± 14.1	41.52 ± 0.013	2.01 ± 0.06	0.29 ± 0.021	8.16 ± 0.410	2.03 ± 0.06
22	1091 ± 0.0	2170 ± 14.1	41.64 ± 0.00	0.68 ± 0.01	0.43 ± 0.007	32.19 ± 0.926	0.80 ± 0.00
23	972 ± 2.8	1902 ± 39.6	41.34 ± 0.00	1.94 ± 0.01	1.09 ± 0.021	29.25 ± 0.552	2.22 ± 0.00
24	966.5 ± 13.4	1974 ± 8.5	41.72 ± 0.01	1.73 ± 0.01	0.99 ± 0.000	29.83 ± 0.219	1.99 ± 0.01
25	1177 ± 7.1	2385 ± 7.1	41.85 ± 0.00	2.38 ± 0.01	0.21 ± 0.007	4.97 ± 0.071	2.39 ± 0.01
26	1277 ± 2.8	2530 ± 14.1	41.10 ± 0.00	0.66 ± 0.00	0.76 ± 0.007	48.00 ± 0.212	0.99 ± 0.01
27	1132 ± 4.2	2345 ± 7.1	42.39 ± 0.00	0.84 ± 0.01	0.19 ± 0.007	12.55 ± 0.170	0.86 ± 0.01
28	1119 ± 2.8	2215 ± 21.2	41.39 ± 0.00	1.01 ± 0.01	0.21 ± 0.007	11.79 ± 0.431	1.03 ± 0.01
29	1292.5 ± 7.8	2615 ± 7.1	41.12 ± 0.00	1.48 ± 0.01	0.34 ± 0.007	12.77 ± 0.205	1.52 ± 0.01
30	1212.5 ± 3.5	2385 ± 21.2	41.41 ± 0.00	1.42 ± 0.00	0.20 ± 0.014	8.05 ± 0.382	1.44 ± 0.01
